# Persistently Elevated Gamma-Glutamyl Transferase and Hepatobiliary Malignancies: A Real-World Cohort Study

**DOI:** 10.3390/cancers18101512

**Published:** 2026-05-08

**Authors:** Arkadeep Dhali, Dushyant Singh Dahiya, Abdul Rafae Faisal, Asad Zaman, Fayaz Khan, Jyotirmoy Biswas, Hareesha Rishab Bharadwaj, Saikat Mandal

**Affiliations:** 1 Academic Unit of Gastroenterology, Sheffield Teaching Hospitals NHS Foundation Trust, Sheffield S5 7AU, UK; 2 School of Medicine, Dentistry and Biomedical Sciences, Queen’s University Belfast, Belfast BT9 7BL, UK; 3 Division of Gastroenterology, Hepatology & Motility, The University of Kansas School of Medicine, Kansas City, KS 66160, USA; 4 Department of General Medicine, CMH Multan Institute of Medical Sciences, Multan 60010, Pakistan; 5 Roswell Park Comprehensive Cancer Center, Buffalo, NY 14263, USA; 6 Barasat Government Medical College and Hospital, Kolkata 700124, India; 7 University Hospitals of North Midlands NHS Trust, Stoke-on-Trent ST4 6QG, UK; 8 School of Medicine, University of Nottingham, Nottingham NG7 2UH, UK

**Keywords:** gamma-glutamyl transferase, cholangiocarcinoma, hepatocellular carcinoma, pancreatitis, cohort

## Abstract

Background: Gamma-glutamyl transferase (GGT) has been associated with increased risks of multiple cancers and mortality. Because GGT is also a marker of hepatobiliary injury, metabolic dysfunction, and alcohol exposure, this study evaluated associations between persistently elevated GGT and hepatobiliary and pancreatic outcomes. Materials and Methods: This retrospective cohort study used TriNetX data from the US Collaborative Network to compare adults aged 40 years or older with persistently elevated GGT against those with normal GGT using a 65 U/L threshold on two occasions 6–12 months apart. Outcomes were assessed from 180 to 1095 days after the index event. Following 1:1 propensity score matching, 14,590 patients per cohort were analyzed. Cox proportional hazards analysis estimated hazard ratios (HRs) with 95% confidence intervals (CIs). Results: Elevated GGT was associated with increased hazards of cholangiocarcinoma (HR 3.715, 95% CI 1.899–7.268), hepatocellular carcinoma (HR 2.260, 95% CI 1.677–3.045), acute pancreatitis (HR 3.359, 95% CI 2.284–4.941), chronic pancreatitis (HR 3.086, 95% CI 1.975–4.821), pancreatic cysts (HR 2.160, 95% CI 1.493–3.127), hospitalization (HR 1.363, 95% CI 1.236–1.503), and all-cause mortality (HR 2.250, 95% CI 2.051–2.467). No statistically significant association was observed with pancreatic cancer (HR 1.591, 95% CI 0.766–3.303; log-rank *p* = 0.209). Mean follow-up after matching was 799.722 ± 391.071 days in the elevated GGT cohort and 844.685 ± 374.676 days in controls. Conclusions: Persistent GGT elevation was associated with hepatobiliary malignancies, pancreatic inflammatory diseases, pancreatic cysts, hospitalization, and all-cause mortality, but not pancreatic cancer. These results suggest that persistent GGT elevation may be a non-specific clinical marker of underlying hepatobiliary and metabolic risk.

## Simple Summary

Gamma-glutamyl transferase is a blood enzyme that can be elevated in people with liver, bile duct, alcohol-related, or metabolic health problems. It is unclear whether a persistently raised level of this enzyme is linked to later pancreatic or liver-related diseases. In this study, the authors compared adults with repeatedly raised gamma-glutamyl transferase levels with similar adults whose levels remained normal, using a large real-world health record database. The aim was to assess whether persistent elevation was associated with pancreatic cancer, liver and bile duct cancers, pancreatitis, pancreatic cysts, hospital admission, and death. The study found stronger associations with liver and bile duct cancers, pancreatitis, pancreatic cysts, hospitalization, and death, but not with pancreatic cancer. These findings may help researchers design future studies to understand whether this enzyme can contribute to broader risk assessment for liver, bile duct, and pancreatic diseases.

## 1. Introduction

Pancreatic cancer remains one of the most lethal malignancies, with a 5-year survival rate of less than 10%, largely attributable to advanced stage at diagnosis [1]. Identifying biomarkers that can stratify risk and facilitate earlier detection represents a critical unmet clinical need. Gamma-glutamyl transferase (GGT), a membrane-bound enzyme involved in glutathione metabolism, has been associated with systemic oxidative stress and hepatobiliary dysfunction across diverse patient populations [2]. Elevated serum GGT levels have been associated with increased risks of multiple cancers, cardiovascular disease, metabolic syndrome, and all-cause mortality in observational cohorts [3,4].

GGT catalyzes the extracellular cleavage of glutathione, facilitating cysteine recovery for intracellular glutathione synthesis and cellular redox homeostasis [5]. Beyond its physiological role, GGT-mediated catabolism of glutathione may generate reactive oxygen species and free iron, creating a pro-oxidant microenvironment that has been linked to oxidative damage and genomic instability in cellular models [6]. Tumor cells in various malignancies frequently overexpress GGT, a characteristic that has been associated with resistance to oxidative stress and chemotherapeutic agents in laboratory investigations [7,8].

Epidemiological evidence has documented associations between elevated GGT and various malignancies. The UK Biobank study, which followed 421,032 participants, identified 586 pancreatic cancer cases and reported associations between baseline GGT quartiles and pancreatic cancer incidence, with the highest GGT quartile showing elevated risk compared to the lowest [9]. Similarly, a Korean nationwide cohort study of patients with diabetes described associations between GGT levels and risks of pancreatic cancer, cholangiocarcinoma, and gallbladder cancer across GGT quartiles [10]. In hepatobiliary malignancies, elevated GGT has been documented in patients with hepatocellular carcinoma (HCC) and has been associated with tumor characteristics and clinical outcomes in observational series [11].

Beyond hepatobiliary cancers, elevated GGT has been associated with inflammatory pancreatic diseases. A Mendelian randomization study suggested a relationship between genetically predicted GGT levels and acute pancreatitis risk [12]. Genetic variants in the GGT1 gene have been associated with chronic pancreatitis susceptibility in observational studies [13], and elevated GGT has been reported in association with pancreatic cystic neoplasms in large cohorts [14]. These observations collectively suggest that GGT may be associated across the spectrum of pancreatic and hepatobiliary pathology.

Most prior studies evaluating GGT and cancer risk have assessed enzyme levels at a single baseline time point, which may not distinguish between transient and sustained elevations. A sustained elevation of GGT might more reliably reflect chronic metabolic derangement, alcohol exposure, cholestasis, or hepatobiliary pathology compared with isolated measurements. Additionally, evaluation of persistent GGT elevation across the spectrum of hepatobiliary malignancy, pancreatic inflammatory disease, pancreatic cystic disease, and pancreatic cancer has been limited in large, propensity-matched cohorts with adjustment for measured demographic, comorbidity, and liver biochemical covariates.

The specific objectives were: (1) to evaluate the association between persistently elevated GGT and incident pancreatic cancer in a large, propensity-matched cohort, (2) to examine associations between elevated GGT and hepatobiliary malignancies and pancreatic inflammatory diseases, and (3) to assess the association between elevated GGT and all-cause mortality. We hypothesized that persistent GGT elevation would be associated with increased incidence of hepatobiliary cancers and pancreatic inflammatory diseases as a marker of chronic hepatobiliary dysfunction, metabolic derangement, or alcohol-related disease burden, rather than as proof of an independent causal effect of GGT. This cohort study has been reported in line with the STROBE guidelines [15].

## 2. Methods

### 2.1. Data Source and Study Design

This multi-institutional retrospective cohort study utilized the TriNetX analytics platform. TriNetX is a global federated health research network providing access to de-identified electronic medical records, including diagnoses, procedures, medications, laboratory values, genomic information, and demographic data from participating healthcare organizations.

### 2.2. Study Population and Cohort Definition

Elevated GGT Cohort (Cohort 1): The exposed cohort consisted of adult patients aged 40 years or older with persistently elevated serum gamma-glutamyl transferase (GGT) levels. In this study, the elevated GGT cohort required GGT in serum or plasma of at least 65.00 U/L, with a confirmatory elevated measurement occurring within 6 months to 1 year after the initial elevated measurement. The final elevated GGT query cohort included 26,136 patients.

Normal GGT Cohort (Cohort 2): The comparison cohort included patients aged 40 years or older with normal GGT levels. GGT in serum or plasma of at most 65.00 U/L, with a confirmatory normal measurement occurring within 6 months to 1 year after the initial normal measurement. The final normal GGT query cohort included 97,649 patients.

Exclusion Criteria: At cohort definition, patients were excluded if acute pancreatitis, pseudocyst of pancreas, cyst of pancreas, benign neoplasm of pancreas, or malignant neoplasm of pancreas occurred on or before the qualifying GGT criterion. For the outcome analyses, patients with the outcome prior to the time window were excluded, and this was applied separately to each outcome. Therefore, patients were removed from a specific outcome analysis if that outcome was already recorded before the start of the outcome window.

### 2.3. Baseline Characteristics

Baseline demographic and clinical characteristics were assessed at the time of the index event and included age at index, sex, and race. Comorbidity burden was assessed for essential hypertension, diabetes mellitus, chronic kidney disease, chronic ischemic heart disease, heart failure, chronic obstructive pulmonary disease, disorders of lipoprotein metabolism and other lipidemias, overweight and obesity, tobacco use, alcohol-related disorders, chronic viral hepatitis, fibrosis and cirrhosis of the liver, and cholecystitis.

Laboratory parameters measured at baseline included alanine aminotransferase (ALT), aspartate aminotransferase (AST), and alkaline phosphatase (ALP), all measured in enzymatic activity per volume (U/L) in serum, plasma, or blood.

### 2.4. Propensity Score Matching

To balance measured baseline covariates between the two cohorts and reduce measured selection bias, 1:1 propensity score matching (PSM) was performed using the listed baseline characteristics. Variables included in the propensity score model were age at index, sex, race, baseline comorbidities (hypertension, diabetes mellitus, chronic kidney disease, chronic ischemic heart disease, heart failure, chronic obstructive pulmonary disease, disorders of lipoprotein metabolism and other lipidemias, overweight and obesity, tobacco use, alcohol-related disorders, chronic viral hepatitis, fibrosis and cirrhosis of the liver, and cholecystitis), and baseline liver biochemical values (ALT, AST, and ALP). PSM does not address unmeasured or incompletely coded confounders, including quantitative alcohol exposure, viral hepatitis serology beyond coded chronic viral hepatitis, liver fibrosis stage, longitudinal metabolic control, or medication exposures.

Characteristics before matching were reported for 16,867 patients in the elevated GGT cohort and 56,517 patients in the normal GGT cohort; after matching, 14,590 patients were retained in each cohort. The success of propensity score matching was evaluated using standardized mean differences (SMD), with SMD < 0.1 considered indicative of adequate balance between groups.

### 2.5. Primary Outcomes and Outcome Definitions

The primary outcomes of interest were incident diagnoses following the index event, assessed using ICD-10-CM diagnostic codes and oncology-specific coding systems. Outcomes were defined as follows.

1. Pancreatic Cancer
2. Cholangiocarcinoma: Intrahepatic bile duct carcinoma or intrahepatic bile duct
3. Hepatocellular Carcinoma (HCC)
4. Pancreatic Cyst
5. Acute Pancreatitis
6. Chronic Pancreatitis
7. Benign Pancreatic Neoplasm
8. All-Cause Mortality
9. Healthcare Utilization: Hospitalization and outpatient visits

### 2.6. Time Window and Follow-Up

The time window for outcome assessment started 180 days after the first occurrence of the index event and ended 1095 days after the first occurrence of the index event. Patients with outcomes occurring prior to the beginning of this outcome window were excluded from the corresponding outcome-specific analysis. Because the analysis window began 180 days after the index event, outcomes recorded between the index date and day 180 were not counted in the reported outcome analyses.

### 2.7. Statistical Analysis

Descriptive Statistics: Baseline characteristics of both cohorts were summarized before and after propensity score matching. Continuous variables were presented as mean ± standard deviation, and categorical variables as frequencies and percentages. The balance of covariates after matching was assessed using standardized mean differences, with SMD < 0.1 indicating successful matching.

Survival Analysis: Time-to-event outcomes were analyzed using Kaplan–Meier survival analysis with log-rank tests to compare cumulative incidence between cohorts.

Hazard Ratios: Cox proportional hazards analyses were used to estimate hazard ratios (HRs) and 95% confidence intervals (CIs) for each outcome, comparing the elevated GGT cohort to the normal GGT cohort. Hazard ratios are reported as measures of association in this observational study and should not be interpreted as causal estimates. TriNetX also reported a proportionality test with chi-square statistic and *p*-value for each estimable hazard ratio.

Follow-up Time: Mean, standard deviation, median, and interquartile range of follow-up time (in days) were calculated for both cohorts before and after propensity score matching.

Statistical Software: All analyses were conducted using the TriNetX online analytics platform. Statistical significance was defined as a two-sided *p*-value < 0.05.

## 3. Results

### 3.1. Study Population and Baseline Characteristics

A total of 26,136 patients met the inclusion criteria for the elevated GGT cohort, while 97,649 patients were identified for the normal GGT comparison cohort. Before propensity score matching, characteristics were reported for 16,867 patients in the elevated GGT cohort and 56,517 patients in the normal GGT cohort. Following 1:1 propensity score matching, 14,590 patients from each cohort were retained and included in the matched outcome analyses.

### 3.2. Characteristics After Propensity Score Matching

**Figure 1:**
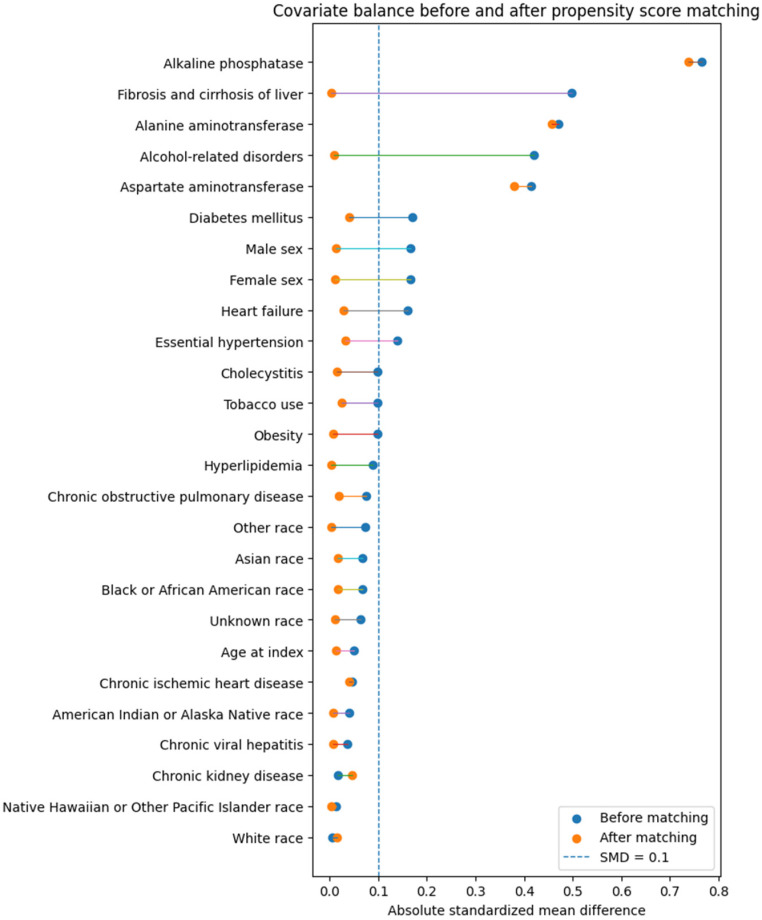
Love plot showing absolute standardized mean differences for baseline covariates before and after propensity score matching.

Propensity score matching reduced measured baseline differences between the two cohorts (Table 1 and Table 2, Figure 1), although liver biochemical values remained higher in the elevated GGT group. Mean age was 57.2 ± 12.7 years in the elevated GGT cohort and 57.4 ± 13.0 years in the normal GGT cohort (*p* = 0.298, SMD = 0.012). Sex distribution was also balanced, with 45.4% females in the elevated GGT group versus 44.8% in controls (*p* = 0.335, SMD = 0.011). Racial distribution was similar across groups, with SMD values ≤ 0.017 for all listed race categories.

All non-laboratory baseline covariates were balanced after matching, with SMD values <0.1. Essential hypertension prevalence was 52.7% versus 51.0% (*p* = 0.006, SMD = 0.032), diabetes mellitus 29.8% versus 28.0% (*p* = 0.001, SMD = 0.040), heart failure 12.0% versus 11.1% (*p* = 0.016, SMD = 0.028), disorders of lipoprotein metabolism and other lipidemias 44.2% versus 44.0% (*p* = 0.741, SMD = 0.004), overweight/obesity 23.2% versus 23.5% (*p* = 0.552, SMD = 0.007), alcohol-related disorders 17.4% versus 17.8% (*p* = 0.461, SMD = 0.009), chronic viral hepatitis 11.5% versus 11.2% (*p* = 0.531, SMD = 0.007), and fibrosis/cirrhosis of the liver 25.8% versus 26.0% (*p* = 0.810, SMD = 0.003). ALT, AST, and ALP remained higher in the elevated GGT cohort after matching.

#### Follow-Up Duration

Mean follow-up time was 799.722 ± 391.071 days (median 1095 days, IQR 640 days) in the elevated GGT cohort and 844.685 ± 374.676 days (median 1095 days, IQR 507 days) in the normal GGT cohort.

### 3.3. Hepatobiliary and Pancreatic Malignancies

Patients with elevated GGT had a higher observed incidence of cholangiocarcinoma compared with those with normal GGT levels. After excluding 150 patients from the elevated GGT cohort and 21 patients from the normal GGT cohort who had cholangiocarcinoma before the outcome window, 14,440 and 14,569 patients remained at risk, respectively. During the outcome window, 38 cholangiocarcinoma events occurred in the elevated GGT cohort versus 11 events in the normal GGT cohort, corresponding to reported risks of 0.003 and 0.001, respectively. The risk difference was 0.002 (95% CI 0.001–0.003; *p* < 0.001), risk ratio was 3.485 (95% CI 1.782–6.816), and odds ratio was 3.492 (95% CI 1.784–6.834).

Kaplan–Meier survival analysis showed survival probabilities at the end of the outcome window of 99.62% in the elevated GGT group versus 99.90% in the normal GGT group (log-rank chi-square = 16.925, df = 1, *p* < 0.001). The hazard ratio for cholangiocarcinoma was 3.715 (95% CI 1.899–7.268). The proportionality test did not show statistically significant deviation (chi-square = 0.814, df = 1, *p* = 0.367).

A higher observed incidence of hepatocellular carcinoma was also identified in the elevated GGT cohort. After excluding patients with pre-existing HCC before the outcome window (783 from the elevated GGT cohort and 408 from the normal GGT cohort), 13,807 and 14,182 patients remained at risk. The elevated GGT cohort experienced 133 HCC events compared with 64 events in the normal GGT cohort, corresponding to reported risks of 0.010 and 0.005, respectively. The risk difference was 0.005 (95% CI 0.003–0.007; *p* < 0.001), risk ratio was 2.135 (95% CI 1.586–2.873), and odds ratio was 2.146 (95% CI 1.591–2.894).

Survival probability at the end of the outcome window was 98.65% in the elevated GGT group versus 99.39% in controls (log-rank chi-square = 30.343, df = 1, *p* < 0.001). The hazard ratio for HCC was 2.260 (95% CI 1.677–3.045). The proportionality test did not show statistically significant deviation (chi-square = 0.493, df = 1, *p* = 0.483).

The association between elevated GGT and pancreatic cancer did not reach statistical significance. After excluding 10 patients with pre-existing pancreatic cancer from the elevated GGT cohort and 0 patients from the normal GGT cohort, 14,580 and 14,590 patients were analyzed, respectively. Pancreatic cancer occurred in 18 patients with elevated GGT versus 12 patients with normal GGT. The reported risk was 0.001 in both cohorts. The risk difference was 0.000 (95% CI −0.000 to 0.001; *p* = 0.272), risk ratio was 1.501 (95% CI 0.723–3.115), and odds ratio was 1.502 (95% CI 0.723–3.119).

Survival probabilities at the end of the outcome window were 99.83% and 99.89%, respectively (log-rank chi-square = 1.580, df = 1, *p* = 0.209). The hazard ratio was 1.591 (95% CI 0.766–3.303), with confidence intervals crossing unity, indicating no statistically significant association. The proportionality test did not show statistically significant deviation (chi-square = 0.218, df = 1, *p* = 0.641). Kaplan–Meier curves showed overlapping trajectories between groups throughout follow-up.

### 3.4. Other Pancreatic Pathology

#### 3.4.1. Benign Pancreatic Neoplasm

Benign pancreatic neoplasm occurred in 11 of 14,590 patients in the elevated GGT cohort and in 0 of 14,588 patients in the normal GGT cohort, after excluding 0 and 10 patients with the outcome prior to the time window, respectively. The reported risks were 0.001 and 0, respectively. The risk difference was 0.001 (95% CI 0.000–0.001; *p* = 0.001). Risk ratio, odds ratio, and hazard ratio were not estimable because no events occurred in the normal GGT cohort. Kaplan–Meier analysis showed a statistically significant difference by log-rank testing (chi-square = 11.687, df = 1, *p* = 0.001), with survival probabilities at the end of the outcome window of 99.89% and 100.00%, respectively.

#### 3.4.2. Pancreatic Cyst

Patients with elevated GGT had a higher observed incidence of subsequent pancreatic cysts. After excluding patients with pancreatic cysts prior to the outcome window (24 from the elevated GGT cohort and 17 from the normal GGT cohort), 14,566 and 14,573 patients, respectively, remained at risk. Pancreatic cysts occurred in 85 patients in the elevated GGT cohort versus 42 patients in the normal GGT cohort, corresponding to reported risks of 0.006 and 0.003, respectively. The risk difference was 0.003 (95% CI 0.001–0.004; *p* < 0.001), the risk ratio was 2.025 (95% CI 1.400–2.928), and the odds ratio was 2.031 (95% CI 1.402–2.941). Kaplan–Meier analysis showed survival probabilities at the end of the outcome window of 99.17% versus 99.62% (log-rank chi-square = 17.517, df = 1, *p* < 0.001), with a hazard ratio of 2.160 (95% CI 1.493–3.127). The proportionality test did not show statistically significant deviation (chi-square = 0.012, df = 1, *p* = 0.913).

#### 3.4.3. Acute Pancreatitis

An association was observed between elevated GGT and acute pancreatitis. After excluding 32 patients from the elevated GGT cohort and 15 patients from the normal GGT cohort with acute pancreatitis before the outcome window, 14,558 and 14,575 patients remained at risk, respectively. Acute pancreatitis occurred in 107 patients in the elevated GGT cohort compared with 34 patients in the normal GGT cohort, corresponding to reported risks of 0.007 and 0.002, respectively. The risk difference was 0.005 (95% CI 0.003–0.007; *p* < 0.001), the risk ratio was 3.151 (95% CI 2.144–4.631), and the odds ratio was 3.167 (95% CI 2.151–4.661). Kaplan–Meier analysis showed survival probabilities at the end of the outcome window of 98.96% versus 99.70% (log-rank chi-square = 42.733, df = 1, *p* < 0.001), with a hazard ratio of 3.359 (95% CI 2.284–4.941). The proportionality test did not show statistically significant deviation (chi-square = 1.065, df = 1, *p* = 0.302).

#### 3.4.4. Chronic Pancreatitis

Elevated GGT was also associated with increased risk of chronic pancreatitis. After excluding 171 patients from the elevated GGT cohort and 131 patients from the normal GGT cohort with pre-existing chronic pancreatitis before the outcome window, 14,419 and 14,459 patients, respectively, remained at risk. Chronic pancreatitis occurred in 75 patients in the elevated GGT cohort versus 26 patients in the normal GGT cohort, corresponding to reported risks of 0.005 and 0.002, respectively. The risk difference was 0.003 (95% CI 0.002–0.005; *p* < 0.001), the risk ratio was 2.893 (95% CI 1.853–4.516), and the odds ratio was 2.903 (95% CI 1.857–4.537). Kaplan–Meier analysis showed survival probabilities at the end of the outcome window of 99.27% versus 99.75% (log-rank chi-square = 27.217, df = 1, *p* < 0.001), with a hazard ratio of 3.086 (95% CI 1.975–4.821). The proportionality test did not show statistically significant deviation (chi-square = 1.446, df = 1, *p* = 0.229).

### 3.5. Mortality and Healthcare Utilization

#### 3.5.1. All-Cause Mortality

Patients with elevated GGT experienced increased all-cause mortality. After excluding 866 patients from the elevated GGT cohort and 385 patients from the normal GGT cohort with mortality recorded before the outcome window, 13,724 and 14,205 patients were analyzed, respectively. During the outcome window, 1387 deaths occurred in the elevated GGT cohort compared with 668 deaths in the normal GGT cohort. The reported risks were 0.101 and 0.047, respectively. The risk difference was 0.054 (95% CI 0.048–0.060; *p* < 0.001), the risk ratio was 2.149 (95% CI 1.966–2.350), and the odds ratio was 2.278 (95% CI 2.071–2.507).

Median survival was not reached in either cohort during the outcome window. Survival probability at the end of the outcome window was lower in the elevated GGT cohort than in the normal GGT cohort (86.95% versus 93.95%; log-rank chi-square = 312.985, df = 1, *p* < 0.001). The hazard ratio for all-cause mortality was 2.250 (95% CI 2.051–2.467). The proportionality test did not show statistically significant deviation (chi-square = 0.540, df = 1, *p* = 0.463).

#### 3.5.2. Hospitalization

After excluding 7020 patients from the elevated GGT cohort and 6414 patients from the normal GGT cohort with hospitalization prior to the outcome window, 7570 and 8176 patients remained at risk, respectively. Hospitalization occurred in 892 patients with elevated GGT versus 736 controls, corresponding to reported risks of 0.118 and 0.090, respectively. The risk difference was 0.028 (95% CI 0.018–0.037; *p* < 0.001), the risk ratio was 1.309 (95% CI 1.193–1.436), and the odds ratio was 1.350 (95% CI 1.218–1.497). Median survival was not reached in either cohort. Survival probability at the end of the outcome window was 84.66% versus 88.52% (log-rank chi-square = 39.009, df = 1, *p* < 0.001), with a hazard ratio of 1.363 (95% CI 1.236–1.503). The proportionality test did not show statistically significant deviation (chi-square = 0.023, df = 1, *p* = 0.881).

#### 3.5.3. Outpatient Visits

After excluding patients with outpatient visits prior to the outcome window (13,026 patients from the elevated GGT cohort and 12,970 from the normal GGT cohort), 1564 and 1620 patients remained at risk, respectively. Outpatient visits occurred in 289 patients in the elevated GGT group compared with 304 controls. No significant difference was detected between groups. The reported risks were 0.185 and 0.188, respectively; the risk difference was −0.003 (95% CI −0.030 to 0.024; *p* = 0.835), the risk ratio was 0.985 (95% CI 0.852–1.139), and the odds ratio was 0.981 (95% CI 0.821–1.173). Kaplan–Meier analysis showed survival probabilities at the end of the outcome window of 75.64% versus 77.12% (log-rank chi-square = 1.129, df = 1, *p* = 0.288), with a hazard ratio of 1.091 (95% CI 0.929–1.282). The proportionality test did not show statistically significant deviation (chi-square = 1.074, df = 1, *p* = 0.300).

## 4. Discussion

This study of 29,180 patients documented that persistently elevated serum GGT was associated with increased incidence of cholangiocarcinoma, hepatocellular carcinoma, acute pancreatitis, chronic pancreatitis, pancreatic cysts, hospitalization, and all-cause mortality. No statistically significant association was observed between elevated GGT and incident pancreatic cancer. These findings describe different patterns of association between persistently elevated GGT and various hepatobiliary and pancreatic outcomes, but they do not establish GGT as an independent causal risk factor.

Interpretation of these findings requires particular caution because GGT is not a disease-specific biomarker. It may reflect cholestasis, hepatic steatosis, alcohol-related enzyme induction, metabolic dysfunction, medication exposure, or established liver disease. Therefore, the observed associations are most appropriately viewed as clinically descriptive and hypothesis-generating. They may identify a group with higher burden of hepatobiliary and metabolic disease, but they should not be interpreted as demonstrating that GGT has an independent causal role in hepatobiliary carcinogenesis or pancreatic inflammation.

### 4.1. GGT and Hepatobiliary Malignancies

The associations observed between elevated GGT and both cholangiocarcinoma and hepatocellular carcinoma are consistent with previous observational reports in the literature. In an analysis of HCC patients undergoing liver transplantation, elevated serum GGT was present in a larger proportion of patients with advanced tumors compared to those with small tumors and was associated with tumor characteristics including microvascular invasion and increased tumor diameter [11]. These observations from clinical series align with the higher incidence of HCC observed in our cohort with persistently elevated GGT, while remaining compatible with GGT acting as a marker of underlying liver disease activity rather than a direct oncogenic exposure.

For cholangiocarcinoma, our findings are comparable to those reported in other observational cohorts. In patients with hepatitis B virus-associated intrahepatic cholangiocarcinoma, elevated preoperative GGT levels were documented in association with adverse postoperative outcomes in an Asian tertiary care series [16]. The magnitude of association with cholangiocarcinoma in our study (HR 3.715) exceeds that reported in the Korean diabetes cohort (HR 1.52 for highest versus lowest GGT quartile) [10], potentially due to differences in study populations, measurement methodology, or our requirement for persistent GGT elevation.

The biological correlates of elevated GGT in hepatobiliary malignancies have been explored in laboratory and clinical investigations. Hepatocytes and biliary epithelial cells express high levels of GGT as part of normal physiology, and enzyme activity increases in response to cholestasis, inflammation, and oxidative stress [17]. In observational studies, tumor cells in several cancers have been noted to express GGT at higher levels than those in surrounding non-malignant tissue, and this expression pattern has been associated with more aggressive tumor phenotypes in cellular investigations [11,18]. The generation of reactive oxygen species through GGT-mediated glutathione catabolism has been characterized in laboratory models as a potential mechanism linking enzyme activity to cellular damage [6].

Elevated GGT is frequently observed in patients with non-alcoholic fatty liver disease (NAFLD), metabolic syndrome, alcohol exposure, cholestasis, and chronic hepatobiliary injury, conditions also associated with hepatobiliary cancer risk [19,20,21,22]. While our propensity score matching balanced the groups on baseline diabetes, obesity, hyperlipidemia, documented alcohol-related disorders, coded chronic viral hepatitis, fibrosis/cirrhosis of the liver, ALT, AST, and ALP, unmeasured aspects of metabolic dysfunction, alcohol dose, viral hepatitis serology and activity, cirrhosis etiology and severity, fibrosis stage, hepatic decompensation, or hepatic steatosis may have influenced the observed associations. The requirement for two GGT measurements enabled a focus on individuals with sustained elevations, which may more accurately reflect underlying chronic hepatobiliary pathology compared to single-point measurements.

The absence of a statistically significant association between elevated GGT and pancreatic cancer in our study is noteworthy, given documented associations in other large observational cohorts. The UK Biobank study, which evaluated 421,032 participants with 586 incident pancreatic cancer cases over a median 7.16-year follow-up, reported an adjusted HR of 1.68 (95% CI 1.22–2.30) comparing the highest to the lowest GGT quartiles [9]. A Korean diabetes cohort also reported a positive association, with HR 1.29 (95% CI 1.22–1.36) for pancreatic cancer in the highest versus lowest GGT quartile [10]. The present finding of HR 1.591 with confidence intervals crossing unity represents a different pattern than these prior reports.

Several factors warrant consideration in interpreting this discordance. First, methodological differences may contribute. Prior studies assessed GGT at baseline only, whereas our requirement for GGT confirmation at two time points separated by 6–12 months selected for individuals with persistent elevations. Longitudinal studies of biomarker trajectories preceding pancreatic cancer diagnosis have documented that liver enzyme abnormalities, including GGT elevation, frequently manifest approximately one year before pancreatic cancer diagnosis, potentially reflecting metabolic alterations or early disease effects [23]. Our study design, intended to capture sustained GGT elevation reflecting chronic pathology, may have inadvertently excluded patients experiencing dynamic enzyme changes during the preclinical disease phase.

Second, the pancreatic cancer outcome in this analysis was based on administrative and oncology coding rather than histology-specific adjudication. Pancreatic cancer has complex metabolic biology, including pathways related to autophagy and nutrient scavenging that have been described in pancreatic cancer models and reviews [24,25]. However, the present TriNetX analysis could not distinguish pancreatic ductal adenocarcinoma from other coded pancreatic malignancies or validate histology, imaging, or pathology. In addition, pancreatic exocrine tissue contributes minimally to circulating GGT levels, which derive predominantly from hepatobiliary sources [17]. Consequently, elevated serum GGT may primarily reflect hepatic and biliary pathology rather than pancreatic-specific pathophysiology.

Third, the relationship between GGT and pancreatic cancer risk in observational studies may be substantially influenced by shared risk factors or intermediate variables. Elevated GGT is strongly associated with NAFLD, metabolic syndrome, and type 2 diabetes, all of which are relevant metabolic contexts in pancreatic cancer epidemiology [26,27]. While our propensity score matching controlled for measured comorbidities, unmeasured confounding from visceral adiposity, severity of insulin resistance, dietary patterns, or glycemic control may obscure or modify associations. Furthermore, reverse causality remains a legitimate concern in observational studies: subclinical pancreatic cancer may induce metabolic alterations, biliary compression from tumor growth, or systemic effects that elevate GGT months to years before clinical diagnosis [23,28]. The parent TriNetX analysis assessed outcomes from 180 to 1095 days after the index event, with mean follow-up after matching of 799.722 days in the elevated GGT cohort and 844.685 days in the normal GGT cohort; this may have been insufficient to fully separate persistent GGT elevation as an exposure from GGT elevation consequent to preclinical disease.

The possibility of non-linear or threshold-dependent associations also warrants consideration. Some observational data suggest that very high GGT levels (e.g., >100 U/L) may carry greater risk associations than moderate elevations [9]. Our dichotomous classification of GGT status (>65 U/L versus ≤65 U/L) may have obscured such non-linear relationships, or the pattern of enzyme elevation relative to other liver enzymes (isolated GGT elevation versus concomitant elevations in alanine aminotransferase (ALT), aspartate aminotransferase (AST), and alkaline phosphatase) may carry differential prognostic significance, reflecting distinct underlying pathologies. Therefore, the current analysis cannot define actionable GGT thresholds or dose–response relationships.

### 4.2. GGT and Pancreatic Inflammatory Diseases

The associations observed between elevated GGT and both acute and chronic pancreatitis are consistent with prior observational and genetic evidence. A Mendelian randomization study employing genetic instruments for GGT levels documented a relationship between genetically predicted GGT and acute pancreatitis risk, suggesting that the association may not be entirely explained by confounding [12]. Additionally, genetic variants in the GGT1 gene have been associated with chronic pancreatitis susceptibility in large genetic association studies [13].

Multiple pathways have been proposed to explain the observed associations between GGT and pancreatitis in observational studies. GGT-mediated generation of reactive oxygen species through glutathione catabolism may potentially contribute to pancreatic acinar cell injury and inflammation, though this mechanism remains incompletely characterized in vivo [29]. Elevated GGT frequently coexists with biliary pathology, including cholelithiasis and biliary sludge, which are well-established causes of acute pancreatitis through mechanical biliary obstruction [30]. Even without overt radiological evidence of biliary obstruction, microscopic changes in bile duct structure or transient elevations in bile duct pressure may be accompanied by GGT elevation, potentially explaining the predictive association with pancreatitis [21]. Chronic alcohol consumption, a major risk factor for pancreatitis, is associated with enzyme induction and oxidative stress mechanisms that increase GGT [22], though our propensity matching on documented alcohol-related disorders was intended to address this confounding.

The finding of elevated GGT in association with subsequent pancreatic cyst (HR 2.160) has limited precedent in the literature. A recent Korean nationwide study reported that higher GGT levels were associated with incident pancreatic cystic neoplasms in a dose–response pattern, though the magnitude of association was more modest (Q4 vs. Q1: HR 1.109) [14]. Pancreatic cysts encompass heterogeneous lesions including serous cystadenomas, mucinous cystic neoplasms, and intraductal papillary mucinous neoplasms (IPMNs). The biological correlates of elevated GGT with pancreatic cyst coding remain unclear in the current literature. Chronic low-grade pancreatic inflammation, oxidative injury to ductal epithelium, or metabolic dysregulation might theoretically influence cyst detection or coding, though these associations would require investigation in dedicated studies.

Benign pancreatic neoplasms were rare, with 11 events in the elevated GGT cohort and no events in the normal GGT cohort. Because the normal GGT cohort had zero events and the hazard ratio was not estimable, this finding should be interpreted cautiously and should not be overemphasized as a primary association.

### 4.3. GGT and Mortality

The two-fold association between elevated GGT and all-cause mortality (HR 2.250) observed in this study is consistent with extensive prior literature documenting GGT as a predictor of mortality across diverse populations. A UK Biobank analysis of approximately 500,000 individuals documented that elevated GGT was associated with all-cause mortality (HR 1.31 for high versus low GGT), with particularly strong associations for liver-related mortality and non-communicable disease-related death [31]. A Korean national cohort similarly reported that individuals in the highest GGT tertile had substantially elevated risk of liver-related mortality [32].

The correlates of elevated GGT and mortality documented in observational studies are likely multifactorial. Elevated GGT reflects hepatic dysfunction and chronic liver pathology, conditions independently associated with adverse outcomes [33]. Elevated GGT has been associated in cross-sectional studies with markers of systemic inflammation such as C-reactive protein and fibrinogen [34], suggesting that GGT elevation may be a marker of chronic inflammation potentially associated with accelerated aging and disease progression. In cancer populations, elevated GGT has been documented in association with tumor stage and clinical outcomes in various malignancy types [35,36], potentially reflecting broader disease burden or aggressive tumor biology.

### 4.4. Study Strengths

This study possesses several methodological strengths. The large multi-institutional cohort derived from the TriNetX platform enabled assessment of several hepatobiliary and pancreatic outcomes, although event counts remained small for some outcomes, particularly pancreatic cancer, cholangiocarcinoma, and benign pancreatic neoplasm. 1:1 propensity score matching improved balance across demographic and comorbidity variables; however, ALT, AST, and ALP remained higher in the elevated GGT cohort, consistent with persistent hepatobiliary biochemical differences between groups. The requirement for persistent GGT elevation confirmed at two time points 6–12 months apart minimizes misclassification from transient GGT elevations due to acute hepatic insults or acute alcohol consumption. The exclusion of patients with pre-existing target outcomes at baseline reduced but did not eliminate, reverse causality related to prevalent or subclinical disease.

### 4.5. Study Limitations

Several important limitations warrant discussion. This retrospective observational cohort study is susceptible to selection bias, residual confounding, reverse causality, and outcome misclassification inherent to research based on large healthcare databases [15,37]. The TriNetX database provides de-identified electronic health records from participating healthcare organizations; therefore, results may not generalize to populations without similar healthcare access or coding practices. Diagnoses coded in electronic health records were not independently validated against medical records, imaging, or pathology specimens in this analysis; cancer outcomes may reflect clinical coding rather than histologically confirmed malignancy.

Despite propensity score matching on measured confounders, unmeasured variables may have influenced observed associations. Important factors not captured include quantitative alcohol consumption patterns, viral hepatitis B and C serology and activity beyond coded chronic viral hepatitis, cirrhosis etiology and severity beyond coded fibrosis/cirrhosis, fibrosis stage, hepatic decompensation, cholestatic liver disease phenotypes, body mass index trajectories over time, glycemic control, medication exposures, dietary composition, and family history of cancer [21,22,26,37]. The ICD-10 code for alcohol-related disorders likely underestimates true alcohol exposure due to underreporting and variable coding practices in clinical settings. Accordingly, the observed associations should be interpreted as associations between persistent GGT elevation and subsequent coded outcomes, not as evidence that GGT itself independently causes hepatobiliary malignancy or pancreatitis.

The potential for reverse causality represents a concern in observational studies of circulating biomarkers and cancer incidence. Subclinical or early-stage malignancies may induce metabolic derangements, systemic effects, weight loss, cholestasis, biliary obstruction, or changes in hepatic function that elevate GGT months to years before clinical diagnosis [23,28]. The parent TriNetX analysis assessed outcomes from 180 to 1095 days after the index event, which excluded events recorded during the first 180 days but did not exclude outcomes occurring after 180 days and within the first 1–2 years after index. Additional sensitivity analyses using longer lag periods, stratifying by follow-up time, or applying time-varying models would strengthen causal inference but were not conducted in the current analysis.

The parent TriNetX output did not report statistically significant proportionality-test deviations for the estimable hazard ratios. However, the use of a single dichotomous GGT threshold limits interpretability because it precludes evaluation of dose–response patterns, sex-specific thresholds, isolated versus cholestatic enzyme patterns, and heterogeneity within the elevated GGT group.

### 4.6. Clinical and Research Implications

The higher observed incidence of cholangiocarcinoma and hepatocellular carcinoma associated with persistent GGT elevation in this study suggests that persistent GGT elevation may warrant attention as part of a broader clinical assessment for underlying hepatobiliary disease. However, absolute event risks were low during the 180- to 1095-day outcome window: 0.003 versus 0.001 for cholangiocarcinoma and 0.010 versus 0.005 for HCC in the elevated and normal GGT cohorts, respectively. GGT is a non-specific marker and should not be used in isolation to initiate cancer surveillance, advanced imaging, or specialist referral. In routine clinical practice, unexplained persistent GGT elevation should instead prompt guideline-consistent evaluation for common hepatic and biliary causes, including alcohol exposure, metabolic dysfunction-associated steatotic liver disease, cholestasis, viral hepatitis where clinically appropriate, medication-related injury, and structural biliary disease [21].

The associations between elevated GGT and both acute and chronic pancreatitis may be clinically relevant when interpreted in the context of symptoms, alcohol exposure, gallstone disease, and other pancreatitis risk factors, but they do not support using GGT alone as a pancreatic disease screening test. The association with pancreatic cyst coding, if confirmed in prospective studies with imaging validation and exclusion of prevalent cysts, might inform future risk models; however, the present study should not be used to change established pancreatic cyst surveillance protocols.

The absence of a significant association between elevated GGT and pancreatic cancer, despite positive findings in other large cohorts, highlights the complexity of biomarker research for pancreatic malignancy. Pancreatic cancer remains exceptionally difficult to detect early, and current clinical practice relies on imaging-based surveillance only for selected high-risk individuals with family history or genetic predisposition [38]. GGT, if incorporated into future risk assessment tools, would likely function as one component within a validated multivariable model rather than as a standalone screening marker.

### 4.7. Future Research Directions

Several investigative approaches could extend the findings from this observational study. Prospective cohort studies incorporating serial GGT measurements at standardized intervals, detailed covariate assessment, and extended follow-up would clarify temporal relationships and evaluate dynamic GGT trajectories as markers of disease incidence. Integration of additional biomarkers reflecting systemic inflammation (C-reactive protein, interleukin-6), oxidative stress (F2-isoprostanes, malondialdehyde), hepatic fibrosis (Fibrosis-4 score, elastography-derived measures), and metabolic function (fasting glucose, insulin, adipokines) would enable more comprehensive characterization of disease-associated phenotypes. Machine learning and unsupervised clustering approaches might identify biosignatures that better predict disease incidence than individual biomarkers.

Laboratory investigations could examine mechanistic pathways linking GGT to observed disease associations. Cell-based and tissue culture models could investigate whether GGT-mediated reactive oxygen species generation contributes to epithelial injury or inflammation in pancreatic and hepatobiliary tissues. Humanized mouse models or patient-derived organoids might characterize the role of GGT in different cellular contexts. Spatial transcriptomics and proteomics of human tumor tissues would clarify localization of GGT expression within malignant and inflammatory lesions.

The relationship between GGT and pancreatic cancer specifically warrants further investigation to explain the discordance with prior cohort studies. Stratified analyses by demographic subgroups, follow-up duration, sex-specific or higher GGT categories, baseline GGT level categories, and enzyme-pattern phenotypes may reveal effect modification not apparent in the primary dichotomous analysis. Investigation of the temporal relationship between GGT elevation and cancer diagnosis, including subclinical phase assessments and lag analyses excluding early events beyond 180 days, could clarify whether dynamic GGT changes during the preclinical disease phase are differentially informative compared to sustained baseline elevations.

Implementation research would be required before any GGT-informed clinical pathway could be recommended. Such work should evaluate whether adding persistent GGT elevation to validated multivariable risk models improves calibration, discrimination, clinical utility, and cost-effectiveness compared with current guideline-based assessment of abnormal liver blood tests and established hepatobiliary or pancreatic surveillance strategies.

## 5. Conclusions

This cohort study documents associations between persistently elevated serum GGT and increased incidence of cholangiocarcinoma, hepatocellular carcinoma, acute pancreatitis, chronic pancreatitis, pancreatic cysts, hospitalization, and all-cause mortality, but not with pancreatic cancer. These findings describe the differential pattern of associations between elevated GGT and various hepatobiliary and pancreatic outcomes in a contemporary cohort. Persistent GGT elevation should be interpreted as a non-specific marker of possible underlying hepatobiliary, metabolic, alcohol-related, or inflammatory disease burden rather than as an independent causal factor or standalone indication for cancer surveillance. Future research incorporating prospective designs, comprehensive biomarker assessment, dose–response analyses, lag analyses, extended follow-up, and mechanistic investigations will be valuable in refining our understanding of GGT across the spectrum of hepatobiliary and pancreatic pathology.

## Figures and Tables

**Table 1:** Baseline Characteristics of Study Cohorts Before Propensity Score Matching.

Characteristic	Elevated GGT (*N* = 16,867)	Normal GGT (*N* = 56,517)	*p*-Value	SMD
**Demographics**				
Age at Index, mean ± SD (years)	57.2 ± 12.6	57.8 ± 14.1	<0.001	0.050
**Sex, ** ***n*** ** (%)**				
Male	8497 (55.5%)	23,652 (47.3%)	<0.001	0.166
Female	6789 (44.4%)	26,329 (52.6%)	<0.001	0.166
**Race, ** ***n*** ** (%)**				
White	10,334 (67.6%)	33,903 (67.8%)	0.579	0.005
Black or African American	2013 (13.2%)	5492 (11.0%)	<0.001	0.067
Asian	764 (5.0%)	3293 (6.6%)	<0.001	0.068
American Indian or Alaska Native	93 (0.6%)	166 (0.3%)	<0.001	0.040
Native Hawaiian or Other Pacific Islander	113 (0.7%)	317 (0.6%)	0.161	0.013
Other Race	532 (3.5%)	1133 (2.3%)	<0.001	0.073
Unknown Race	1448 (9.5%)	5704 (11.4%)	<0.001	0.063
**Comorbidities, ** ***n*** ** (%)**				
Essential Hypertension	8195 (53.6%)	23,359 (46.7%)	<0.001	0.138
Diabetes Mellitus	4685 (30.6%)	11,575 (23.1%)	<0.001	0.169
Chronic Kidney Disease	2586 (16.9%)	8155 (16.3%)	0.081	0.016
Chronic Ischemic Heart Disease	2495 (16.3%)	7348 (14.7%)	<0.001	0.045
Heart Failure	1940 (12.7%)	3902 (7.8%)	<0.001	0.161
COPD	1392 (9.1%)	3545 (7.1%)	<0.001	0.074
Hyperlipidemia	6830 (44.6%)	20,126 (40.2%)	<0.001	0.089
Obesity	3620 (23.7%)	9828 (19.7%)	<0.001	0.098
Tobacco Use	1038 (6.8%)	2252 (4.5%)	<0.001	0.099
Alcohol-Related Disorders	3130 (20.5%)	3203 (6.4%)	<0.001	0.421
Chronic Viral Hepatitis	1757 (11.5%)	5189 (10.4%)	<0.001	0.036
Fibrosis and Cirrhosis of the Liver	4449 (29.1%)	4968 (9.9%)	<0.001	0.498
Cholecystitis	382 (2.5%)	584 (1.2%)	<0.001	0.099
**Laboratory Values, mean ± SD (U/L)**				
Alanine Aminotransferase (ALT)	58.4 ± 88.4	27.3 ± 29.8	<0.001	0.471
Aspartate Aminotransferase (AST)	61.0 ± 113.6	26.9 ± 26.5	<0.001	0.414
Alkaline Phosphatase (ALP)	184.9 ± 184.7	82.1 ± 44.6	<0.001	0.766

Abbreviations: ALP, alkaline phosphatase; ALT, alanine aminotransferase; AST, aspartate aminotransferase; COPD, chronic obstructive pulmonary disease; GGT, gamma-glutamyl transferase; SD, standard deviation; SMD, standardized mean difference.

**Table 2:** Baseline Characteristics of the Study Cohorts After Propensity Score Matching.

Characteristic	Elevated GGT (*N* = 14,590)	Normal GGT (*N* = 14,590)	*p*-Value	SMD
**Demographics**				
Age at Index, mean ± SD (years)	57.2 ± 12.7	57.4 ± 13.0	0.298	0.012
**Sex, ** ***n*** ** (%)**				
Male	7955 (54.5%)	8041 (55.1%)	0.312	0.012
Female	6624 (45.4%)	6542 (44.8%)	0.335	0.011
**Race, ** ***n*** ** (%)**				
White	9862 (67.6%)	9954 (68.2%)	0.249	0.014
Black or African American	1913 (13.1%)	1837 (12.6%)	0.184	0.016
Asian	746 (5.1%)	802 (5.5%)	0.144	0.017
American Indian or Alaska Native	82 (0.6%)	73 (0.5%)	0.469	0.008
Native Hawaiian or Other Pacific Islander	111 (0.8%)	106 (0.7%)	0.733	0.004
Other Race	479 (3.3%)	468 (3.2%)	0.716	0.004
Unknown Race	1397 (9.6%)	1350 (9.3%)	0.346	0.011
**Comorbidities, ** ***n*** ** (%)**				
Essential Hypertension	7682 (52.7%)	7447 (51.0%)	0.006	0.032
Diabetes Mellitus	4351 (29.8%)	4085 (28.0%)	0.001	0.040
Chronic Kidney Disease	2431 (16.7%)	2187 (15.0%)	<0.001	0.046
Chronic Ischemic Heart Disease	2343 (16.1%)	2133 (14.6%)	0.001	0.040
Heart Failure	1751 (12.0%)	1620 (11.1%)	0.016	0.028
COPD	1294 (8.9%)	1216 (8.3%)	0.103	0.019
Hyperlipidemia	6445 (44.2%)	6417 (44.0%)	0.741	0.004
Obesity	3392 (23.2%)	3435 (23.5%)	0.552	0.007
Tobacco Use	936 (6.4%)	848 (5.8%)	0.032	0.025
Alcohol-Related Disorders	2543 (17.4%)	2591 (17.8%)	0.461	0.009
Chronic Viral Hepatitis	1675 (11.5%)	1641 (11.2%)	0.531	0.007
Fibrosis and Cirrhosis of the Liver	3769 (25.8%)	3787 (26.0%)	0.810	0.003
Cholecystitis	314 (2.2%)	284 (1.9%)	0.215	0.015
**Laboratory Values, mean ± SD (U/L)**				
Alanine Aminotransferase (ALT)	58.6 ± 89.0	28.5 ± 28.4	<0.001	0.456
Aspartate Aminotransferase (AST)	60.5 ± 114.2	28.9 ± 30.9	<0.001	0.379
Alkaline Phosphatase (ALP)	184.8 ± 185.9	84.7 ± 46.6	<0.001	0.738

Abbreviations: ALP, alkaline phosphatase; ALT, alanine aminotransferase; AST, aspartate aminotransferase; COPD, chronic obstructive pulmonary disease; GGT, gamma-glutamyl transferase; SD, standard deviation; SMD, standardized mean difference.

## Data Availability

The data used in this study were obtained from the TriNetX US Collaborative Network. Individual-level patient data are not publicly available because TriNetX provides access to de-identified electronic health record data under institutional data-use agreements. Aggregate results generated for this study are included in the article. Further inquiries can be directed to the corresponding author.
